# Healthy communication in the promotion of healthy aging during COVID-19 pandemic

**DOI:** 10.3325/cmj.2020.61.177

**Published:** 2020-04

**Authors:** Marijana Braš, Veljko Đorđević, Neda Pjevač, Ivana Đurić

**Affiliations:** 1 Department of Psychiatry and Psychological Medicine, Clinical Hospital Centre Zagreb, Zagreb, Croatia; 2 Center for Palliative Medicine, Medical Ethics and Communication Skills, University of Zagreb School of Medicine, Zagreb, Croatia; 3 Department of Educational Technology & Educational Multimedia Center, University of Zagreb School of Medicine, Andrija Štampar School of Public Health, Zagreb, Croatia; 4 Zagreb’s Institute for the Culture of Health, Zagreb, Croatia

Communication in medicine is a fundamental clinical skill and one of the most important tools we have for providing quality patient care and improving patient satisfaction. Good communication between health professionals and patients is mutually beneficial and essential in the therapeutic process. The patient can receive a better and more personalized care, while the health worker will find it easier to perform his work. Communication in medicine has evolved from the paternalistic model to the current model of collaborative partnership, which highlights the importance of informed consent and shared decision making. In person-centered medicine and people-centered health care, an effective communication is regarded as the best example of *ars medica* (the art of medicine), while the communication skills training has become internationally accepted as an essential component of medical education.

## Communication with the elderly – how to develop specific communication skills and avoid prejudice

Geriatric medicine requires even more effective and specific communication skills. In the past twenty years, while attending and organizing numerous training courses in social psychiatry, communication skills in medicine, and palliative medicine, we had the privilege to meet some of the world's leading experts in the field of geriatrics and gerontology. Today, we are fully aware how right they were to advocate the improvement of 1) communication skills when dealing with the elderly patients and 2) our personal attitudes toward aging in general and elderly patients. Equally, a great emphasis has been placed on promoting healthy aging and the harmonization of different national strategies and policies targeting the elderly (health, social, demographic). The latter is especially relevant in the context of rapid population aging. A considerable increase in the number of persons who live to a very old age, either relatively healthy or with serious illnesses that require extensive care, calls for new responses and health care strategies.

Aging can bring about unique health issues, and therefore it is important to understand the challenges faced by people as they age. The most common health issues in old age include chronic health issues, cognitive and mental health challenges, the risk of physical injury, sensory impairments, malnutrition, oral health problems, etc. In addition, the elderly often have multiple pathologies. Bearing this in mind, it is not surprising that the medical aspects of aging have been given disproportionate attention. Conversely, aging, including healthy aging, remains insufficiently addressed both in practice and research, while the public, but also medical professionals, often have explicit and implicit prejudice against elderly patients (1).

## Experience from clinical practice reveals major psychosocial burden of aging

Our clinical experience with elderly persons without dementia (usually between 75 and 85 years old) who have sought psychological help in our institution in recent months has shown that the major psychosocial problems afflicting the elderly are loneliness, depression, social isolation, and a general lack of understanding from their social environment. The persons surrounding them fail to recognize that the elderly desire to continue working on themselves, live a life, find a new love, and feel “young and alive” as much as possible. Among the most prominent topics that elderly patients have raised are their new infatuations, sorrows, relationships with children, and the pursuit of new hobbies or jobs. They often report that their doctors are supportive and take sufficient time to hear their wishes and ideas for a better quality of life; but some of them also felt ignored or completely misunderstood. We repeatedly hear that “The doctor focused on my symptom or illness, not on me as a person and my life goals.” or “Everyone around me acts like I have already been written off and obsolete, while I still feel so young and lively.”

Unfortunately, depression in the elderly is often underdiagnosed and undertreated. However, besides psychiatric treatment, depression treatment in the elderly can be facilitated by the improvement of living conditions and social support from the family, friends, or support groups. Many of our elderly patients have expressed a wish to live in a residential community with available support services where they would have their own flat or a small house. In their view, this type of arrangement would allow them to receive optimal health care by trained staff and give them a sense of security that they would not “die alone in their apartment, without anyone knowing.”

## COVID-19 pandemic – a new burden and stigmatization threat to the elderly

The ongoing pandemic of coronavirus disease 2019 (COVID-19), caused by severe acute respiratory syndrome coronavirus 2 (SARS-CoV-2), is undoubtedly the defining global health crisis of our time and the greatest challenge the world has faced since the World War II. It has greatly emphasized the importance of healthy communication with the elderly, but even more so, about them. Following the initial outbreak of COVID-19, misinformation and disinformation emerged regarding its origin, scale, risk factors, prevention, and treatment. The available evidence suggests that older adults are at a significantly increased risk of severe disease following COVID-19 infection because of multimorbidity, decreased immune function, and normal physiological changes associated with aging. When distributing health-related information, it is very important to know how to communicate with the elderly about COVID-19 and how to protect and support them. It is especially important to protect the older people with frailty, dementia, and those who live alone and without social support. The current health challenge is indeed a global test of our humanity and solidarity.

While social distancing and self-isolation measures can help protect the elderly, they, especially mandatory long-term lockdown, seem to be contributing to increased ostracism and public hostility toward older individuals, especially when seen outdoors. It remains to be investigated to what degree these measures will negatively affect the general mental status of the elderly.

Finally, it has to be underlined that COVID-19 does not affect only the elderly, and age seems to be just one among the many risk factors of disease outcome. Therefore, it is very important to spread clear and true information and prevent stigmatization of any social group. In addition, one’s general health state before the infection, and regardless of age, plays a crucial role in disease contraction and development. Therefore, it is more than ever important to highlight, promote, and educate the wider public about the concept of healthy aging.

This is in line with the highly acclaimed concepts of social medicine and the public health legacy left to Croatia and the entire world by professor Andrija Štampar. The ten principles he laid down in 1926 are today more relevant than ever, and it is our strongest belief that these should be revisited, both in practice and in education.

## The person-centered medical interview for elderly patients

As in other medical branches, person-centered medical interview is an essential and very important tool in elderly care (2). The medical interview is an integral part of the comprehensive geriatric assessment and diagnostics. The interview must include a range of biological, psychological, social, and spiritual components. A special emphasis should be placed on specific challenges that we face when communicating with this population. Older people often have functional difficulties (hearing, vision, and even cognitive impairment) that we need to be able to recognize and accommodate for in the interview. One of the challenges is how to deliver bad news to patients with serious diseases. The existing protocols (eg, SPIKES) need to be applied with an understanding of old patients’ specificities (3). When communicating with these patients, it is also important to recognize specific emotional responses of old people to the disease. They are often lonely, have a number of chronic illnesses, greater fear of death, the experience of many losses and tragedies in their life, and fear of helplessness and functional disability that would make them dependent on other people’s care. During the interview, it is very important to differentiate between a “normal” emotional reaction in a specific context (including a cultural context) and a developed, pathological psychiatric disorder requiring treatment. At the same time, one should tackle the prevailing prejudice that “it is normal to be depressed when you are old” or that “it is not normal to talk about love or sexual dysfunction because you are old” (4). Finally, the medical interview has to include the consultation with the patient’s family. Medical practitioners should conduct an interview that will have a high motivational effect on the patient. The interviewer should promote healthy aging – point out the good aspects and encourage healthy life habits – while at the same time explaining to the patient that they can still live a quality life regardless of the numerous illnesses. Furthermore, it is important to inquire about the patient’s greatest problems and to recognize the context in which they live and the fears they experience (5).

Geriatric assessment is interdisciplinary, requiring an effective communication within the medical team. The team must also include the patients and their family, who have to be made equal participants in the communication process. Having recognized this, over the past ten years we have promoted and gradually introduced into the curriculum of the University of Zagreb School of Medicine new modules dedicated to the communication with the elderly (6). These modules have been integrated into several courses at all levels (undergraduate, graduate, and continuous medical training). We have also included patients in the teaching process through the model “patient as a teacher.” This experiential learning method has proven to be an excellent tool and has importantly affected knowledge and attitudes among students and health care professionals (7-9).

## Conclusion

Promoting healthy aging at all levels (from local to global) is a continuous process in which it is essential to improve communication. As one of the most important public health issues today, the issue of aging should not only concern and include directly interested parties (patients, medical practitioners) but also a wider public and especially the media. So far, apart from the anecdotal evidence or a few case reports, healthy aging has attracted little or no interest among Croatian researchers. More comprehensive research is very much needed, especially on the communication competencies (knowledge, attitudes, and skills) of health care professionals and how these competencies are affected by educational interventions.
